# Development and validation of predictive models for myopia onset and progression using extensive 15-year refractive data in children and adolescents

**DOI:** 10.1186/s12967-024-05075-0

**Published:** 2024-03-17

**Authors:** Jing Zhao, Yanze Yu, Yiming Li, Feng Li, Zhe Zhang, Weijun Jian, Zhi Chen, Yang Shen, Xiaoying Wang, Zhengqiang Ye, Chencui Huang, Xingtao Zhou

**Affiliations:** 1 Department of Ophthalmology and Vision Science, Eye and ENT Hospital, Fudan University, 83 Fenyang Road, Shanghai, 200031 China; 2 NHC Key Laboratory of Myopia, Fudan University, Shanghai, China; 3 Laboratory of Myopia, Chinese Academy of Medical Sciences, Shanghai, China; 4 Department of Research Collaboration, R&D Center. Beijing Deepwise & League of PHD Technology Co, Ltd., Beijing, 100080 China; 5 Information Center, Eye & ENT Hospital, Fudan University, Shanghai, 200031 China

**Keywords:** Myopia, High myopia, Cycloplegic refraction, Machine learning, Predictive model

## Abstract

**Background:**

Global myopia prevalence poses a substantial public health burden with vision-threatening complications, necessitating effective prevention and control strategies. Precise prediction of spherical equivalent (SE), myopia, and high myopia onset is vital for proactive clinical interventions.

**Methods:**

We reviewed electronic medical records of pediatric and adolescent patients who underwent cycloplegic refraction measurements at the Eye & Ear, Nose, and Throat Hospital of Fudan University between January 2005 and December 2019. Patients aged 3–18 years who met the inclusion criteria were enrolled in this study. To predict the SE and onset of myopia and high myopia in a specific year, two distinct models, random forest (RF) and the gradient boosted tree algorithm (XGBoost), were trained and validated based on variables such as age at baseline, and SE at various intervals. Outputs included SE, the onset of myopia, and high myopia up to 15 years post-initial examination. Age-stratified analyses and feature importance assessments were conducted to augment the clinical significance of the models.

**Results:**

The study enrolled 88,250 individuals with 408,255 refraction records. The XGBoost-based SE prediction model consistently demonstrated robust and better performance than RF over 15 years, maintaining an *R*^2^ exceeding 0.729, and a Mean Absolute Error ranging from 0.078 to 1.802 in the test set. Myopia onset prediction exhibited strong area under the curve (AUC) values between 0.845 and 0.953 over 15 years, and high myopia onset prediction showed robust AUC values (0.807–0.997 over 13 years, with the 14th year at 0.765), emphasizing the models' effectiveness across age groups and temporal dimensions on the test set. Additionally, our classification models exhibited excellent calibration, as evidenced by consistently low brier score values, all falling below 0.25. Moreover, our findings underscore the importance of commencing regular examinations at an early age to predict high myopia.

**Conclusions:**

The XGBoost predictive models exhibited high accuracy in predicting SE, onset of myopia, and high myopia among children and adolescents aged 3–18 years. Our findings emphasize the importance of early and regular examinations at a young age for predicting high myopia, thereby providing valuable insights for clinical practice.

**Supplementary Information:**

The online version contains supplementary material available at 10.1186/s12967-024-05075-0.

## Background

Myopia, also referred to as nearsightedness, is a growing global epidemic of great concern [1, 2]. Multiple population-based studies have reported an unprecedented “myopia boom” worldwide, especially in East Asia [3]. This is particularly alarming since myopia, especially high myopia (refractive error ≤ − 6.00 diopters), has been associated with a range of vision-threatening complications, including glaucoma and maculopathy, which can lead to irreversible vision loss [4]. These complications can impose a substantial burden on both quality of life and economic productivity, particularly for the young working-age population [5].

The distribution of myopia differs among populations of various racial and environmental backgrounds [6]. The prevalence of childhood myopia in China and Singapore is significantly higher than in European countries, with a discernible trend towards younger onset within those populations [7]. While onset typically occurs at around 5–15 years of age, recent reports highlighted an escalating prevalence of early onset in infants and preschool children [8, 9]. Notably, children with myopia onset during early school ages are at a higher risk of developing high myopia [10, 11]. An avenue for addressing myopia and its complications involves early detection and treatment, emphasizing the significance of timely risk stratification and the implementation of effective prevention strategies.

In optometric and ophthalmic clinics, a gap remains in accurately predicting the onset of myopia and in estimating the likelihood of its advancement to high myopia. Currently, ophthalmologists rely heavily on their clinical experience for tackling inquiries, due to the absence of precise predictive instruments. Consequently, they advocate for periodic refractive examinations for children as a proactive measure to prevent myopia. During annual follow-ups, cycloplegic refraction is the main and routine tool used to evaluate the onset and severity of myopia [12]. Therefore, the ophthalmic and optometric clinics have provided a large-scale dataset of consecutive refractive results.

Despite the availability of long-term refractive development records, extracting valuable insights into the progression of myopia has proven challenging because of potential “noise” and suboptimal regression methods. Therefore, pre-emptive action is necessary to overcome these challenges. Recently, digital healthcare technologies have leveraged the potential of artificial intelligence (AI) to develop adjunctive solutions that offer scalability, portability, and reliability [13]. These techniques supplement traditional epidemiological methods, provide correlation analyses, and leverage intricate interactions among predictors to gain novel insights [14]. In myopia prediction, machine learning approaches have been introduced to predict progression in school-aged children [15], axial length growth in myopic children [16], myopia status in adolescents [17], pathologic myopia detection [18], the risk for developing high myopia [19], and identification of risk factors for disease progression [20]. However, the potential of AI-assisted utilization of cycloplegic refractive data for integrated, long-term predictions of age-specific refractive error status and the onset of myopia and high myopia remains uncertain.

This study endeavors to achieve accurate predictions employing a machine learning algorithm that utilizes longitudinal cycloplegic refractive data. The specific focus is on predicting spherical equivalent (SE) values and discerning the probability of developing myopia and high myopia at designated future time points.

## Methods

### Data collection and ethics statement

The refractive dataset for this study, collected from January 2005 to December 2019 at the Eye & Ear, Nose, and Throat Hospital of Fudan University (FDUEENT), underwent secure extraction with specific criteria and structured queries by collaborating with the Information Center. To prioritize privacy, all datasets were deidentified before transfer to the study investigators, ensuring transparency and reproducibility through detailed documentation. The Institutional Review Board of the FDUEENT approved the study protocol (approval code 2020-10-29), and all procedures adhered to the principles of the Declaration of Helsinki. The cohort characteristics are presented in Table 1. The data primarily comprised patient age and cycloplegic refraction measurements, which were obtained at various time intervals. The cycloplegic SE was calculated by the standard formula: SE = sphere + 1/2 × cylinder.

**Table 1:** Characteristics of the individuals included in the study

Characteristics	Total	Training set	Testing set
Number of persons	88250	52950	35300
Number of records	408255	244953	163302
Male, number (%)	45184 (51.2)	27040 (51.1)	18144 (51.4)
Follow-up, mean ± SD, years	3.4 ± 2.7	3.6 ± 2.1	3.3 ± 1.7
Age at first visit, mean ± SD, years	7.7 ± 4.0	8.1 ± 3.5	7.6 ± 3.7
Age at last visit, mean ± SD, years	14.3 ± 4.6	13.5 ± 4.0	14.5 ± 4.8
SE at first visit, mean ± SD, diopters	0.4 ± 3.5	0.8 ± 2.8	(− 0.1) ± 2.3
SE at last visit, mean ± SD, diopters	(− 1.3) ± 3.6	(− 1.8) ± 3.2	(− 1.2) ± 2.9

### Study setting

This study consisted of two main parts. First, all available records (Study 1) were included and randomly divided into training and testing datasets at a 6:4 ratio. Regression models for predicting age-specific SE were trained using the training dataset and verified using the testing dataset. Second, patients from Study 1 who remained non-myopic (Study 2) and those who did not develop high myopia (Study 3) at the initial examination were assigned to two separate groups to evaluate the onset of myopia and high myopia prediction models. For both groups, the dataset was randomly divided into training and testing sets at a 6:4 ratio.

### Dataset preprocessing

**Fig. 1:**
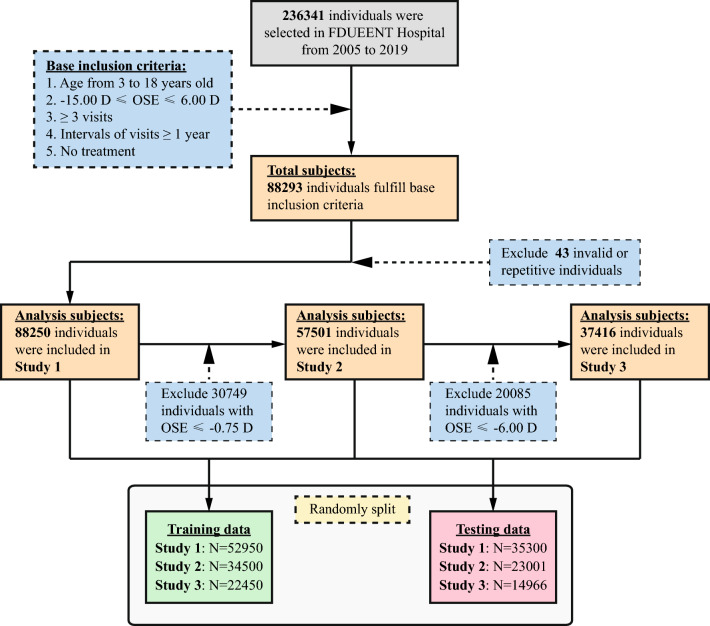
Flowchart of the quality control and preprocessing process for the data set. FDUEENT, Eye & ENT Hospital of Fudan University

A total of 88,293 participants met the inclusion criteria, and the population included individuals aged 3–18 years, with an initial cycloplegic SE of −15.00–6.00 diopters (D), who had follow-up data from at least two visits with an interval of at least one year between consecutive visits. After excluding 43 individuals with invalid or repetitive data, data from 88,250 patients were analyzed. To determine the presence of heterogeneity across different years within our dataset, which spanned approximately 15 years, we conducted 5 year period analyses (2005–2009, 2010–2014, and 2015–2019). A flowchart of the data collection and preprocessing is presented in Fig. 1.

In addressing data quality concerns, several preprocessing steps were implemented. Outlier detection utilizing the Interquartile Range (IQR) method, where values exceeding 1.5 * IQR were re-evaluated, retaining genuine special cases and exclusion for data collection or recording errors. For handling missing values, a direct exclusion approach was adopted due to the study's focus on a small yet crucial set of variables, including age, baseline SE, subsequent follow-up SE, and measurement time. Following the cleaning process, feature normalization was performed before modeling. The Z-Score normalization method was applied, involving the calculation of mean and standard deviation for each variable and application of the Z-Score formula, standardizing the sample mean to zero (μ = 0) and variance to unit (σ = 1). This ensured consistent data scaling during the modeling process, thereby enhancing model stability and interpretability.

The development of myopia or high myopia is a gradual process; therefore, the number of participants who developed myopia or high myopia in the early years after the first examination was lower than that in the normal group. This bias in the training dataset can influence our models to some degree, as some consider the full population in its entirety. To address this issue, we employed a pipeline for oversampling and undersampling. Synthetic Minority Over-sampling Technique (SMOTE) and random undersampling methods were used to synthesize new samples for the minority class and delete samples from the majority class, respectively [21]. Furthermore, grid-search method was applied to explore the best resampling rate.

### Predictors and outcomes

In this study, we aimed to develop algorithms to predict refraction values and the onset of myopia and high myopia for 15 years following an initial examination, using age at baseline (AGE), original SE (OSE) at the first examination, and annual myopia progression rate (AMPR) as predictors. AMPR was calculated as follows:

$${\text{AMPR}}=\frac{|\Delta {\text{SE}}|}{|\Delta {\text{age}}|}$$

where ∆age and ∆SE represent differences in age and SE between the first and second visits, respectively. In addition, we incorporated the SE in the year after the first examination (NSE) as a comparative analysis factor in the model inputs. We collected all data from the right eye, considering the high correlation between the eyes.

### Model development and validation

To verify the performance of the different methods, we performed a methodological comparison analysis of the results of the random forest (RF) and gradient boosted tree (XGBoost) algorithms in the context of the SE prediction models. A combination of grid-search and five-fold cross-validation, which randomly split all the samples into five groups, was performed during the hyperparameter selection process in our original training data. Four of the groups (80%) were used as actual training data and one group (20%) was used internal validation data. To further ensure model robustness, each cross-validation process was repeated five times; thus, each model was trained with different hyperparameters for a total of 25 times, based on a different training set each time and the average results of internal validation, as the comparable model selection criteria. We tested the performance of the selected models in the testing group to determine the potential working mechanisms of the algorithm predicting refractive values and the onset of myopia and high myopia over 15 years.

### Model evaluation

To assess the predictive ability of the regressive models for the targeted SE, we calculated the coefficients of determination (${R}^{2}$), mean absolute error (MAE), and mean squared error (MSE). Additionally, for classification performance, we used seven other evaluation metrics: area under the curve (AUC), precision, accuracy, F1_score, sensitivity (recall), specificity, and brier score. In addition, the Shapley-Additive-exPlanations (SHAP) algorithm was applied to quantify the importance of each feature in the models [22].

### Statistical analysis

Continuous variables are presented as mean ± standard deviation (SD). To examine the predictive capabilities across different age groups, considering factors, such as the regularity of refractive development and academic pressure, we conducted age-specific subgroup analyses. Age groups were defined as follows: 3–6 years (preschool students), 7–14 years (primary and junior high school students), and 15–18 years (senior high school students). These subgroup analyses enabled us to evaluate the model performance in the testing group for each age cohort. All data analyses, model constructions, and evaluations were performed using Python (version 3.7.2). The RF and XGBoost models were developed using scikit-learn and XGBoost library, respectively.

## Results

### Study population characteristics

**Fig. 2:**
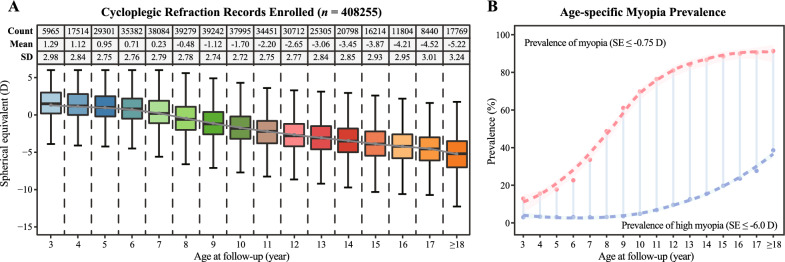
Spherical equivalent distribution and myopia prevalence based on the refraction records enrolled. **A** Spherical equivalent distribution for children and adolescents aged 3 to over 18 years, from 2005 to 2019. **B** Myopia and high myopia prevalence for individuals included in the study aged 3 to over 18 years, from 2005 to 2019

The study included 88,250 participants with 408,255 records (Study 1), of whom 51.2% were males and 48.8% were females. The mean age and SE at baseline of the full study cohort was 7.7 ± 4.0 years and 0.40 ± 3.50 D, respectively. The individuals were randomly divided into an original training and a testing dataset comprising 60% (n = 52,950) and the remaining 40% (n = 35,300) of the data. Further details are listed in Table 1. Figure 2A depicts the age-specific SE distribution based on all the enrolled refraction records in our dataset. The results revealed a gradual decrease in SE with increasing age, which was consistent with the expected pattern of normal growth and development. Figure 2B illustrates the prevalence of myopia and high myopia in the dataset. The year-stratified analysis indicated that there was no significant difference in the age-specific distribution of SE (Additional file 1: Fig. S1A) or the prevalence of myopia and high myopia (Additional file 1: Fig. S1B, C) among the three periods examined. This finding suggests a high level of internal homogeneity in the dataset.

### SE prediction model construction and performance

**Fig. 3:**
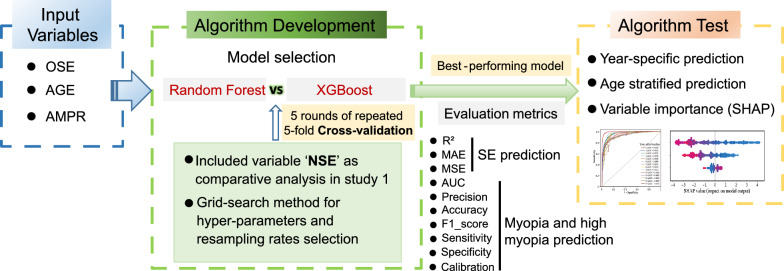
Overall design of model training and performance evaluation

A comprehensive illustration of the model-construction workflow is presented in Fig. 3. By searching the hyperparameters of the models in the original training group using the grid-search method, the best values of the hyperparameters, including maximum depth, learning rate, and number of weak learners (n_estimators), were verified as 2, 0.05, and 150, respectively. The inclusion of three features (AGE, OSE, and AMPR) in the regressive models for predicting SE over a 15 year period resulted in XGBoost attaining higher *R*^*2*^ and lower MAE/MSE values than RF at each prediction time point after the baseline assessment (Table 2). A comparison of the models developed using samples with NSE records to models developed without the NSE records showed that the former exhibited higher prediction performance in most years, especially in the 11–14 years. Notably, the MAE values ranged from 0.078 to 0.720 D for the first nine years following the baseline examination, which was below the clinically acceptable accuracy threshold of 0.75 D, considering the refraction measurement variations [23]. These findings suggest that the XGBoost algorithm outperformed RF in predicting SE, and the requirement of NSE further improved the predictability of SE. The detailed and age-specific performances of the regression models in the test group are presented in Table 2 and Additional file 1: Fig. S2. Additional file 1: Fig. S3 illustrates the distribution of predicted versus actual values of SE. In the initial prediction year, the prediction error fell within 0.15 D for all cases, resulting in a 100% accuracy within this range. In the second prediction year, the error was limited to within 1 D for 89% of the cases.

**Table 2:** Regressive performance of the Random Forest and XGBoost algorithms

Year	NSE required	Model	*R*^2^	Total		3–6 years		7–14 years		15–18 years	
				MAE	MSE	MAE	MSE	MAE	MSE	MAE	MSE
1	No	RF	0.958	0.360	0.364	0.408	0.441	0.331	0.441	0.369	0.343
		XGBoost	0.992	0.078	0.099	0.077	0.112	0.078	0.075	0.091	0.345
2	No	RF	0.920	0.491	0.772	0.563	0.933	0.435	0.633	0.529	0.698
		XGBoost	0.952	0.397	0.657	0.472	0.850	0.332	0.474	0.336	0.489
	Yes		0.949	0.437	0.723	0.499	0.917	0.375	0.504	0.388	1.010
3	No	RF	0.886	0.619	1.176	0.682	1.361	0.581	0.992	0.619	1.484
		XGBoost	0.922	0.577	1.128	0.638	1.379	0.533	0.878	0.585	1.103
	Yes		0.930	0.588	1.063	0.652	1.324	0.527	0.781	0.570	0.880
4	No	RF	0.840	0.720	1.715	0.789	2.055	0.663	1.380	0.850	1.856
		XGBoost	0.896	0.704	1.540	0.758	1.776	0.647	1.282	0.693	1.931
	Yes		0.902	0.693	1.561	0.771	1.789	0.625	1.250	0.613	3.187
5	No	RF	0.829	0.619	1.814	0.733	2.139	0.557	1.487	0.570	2.306
		XGBoost	0.869	0.688	1.929	0.777	2.372	0.635	1.412	0.763	3.349
	Yes		0.889	0.678	1.753	0.767	2.116	0.618	1.357	0.669	1.269
6	No	RF	0.794	0.606	2.235	0.734	2.670	0.515	1.770	0.600	3.054
		XGBoost	0.860	0.679	2.133	0.789	2.508	0.581	1.631	0.935	4.247
	Yes		0.882	0.643	1.925	0.802	2.383	0.505	1.410	0.479	1.718
7	No	RF	0.785	0.554	2.375	0.778	2.968	0.442	1.783	0.394	2.059
		XGBoost	0.841	0.691	2.401	0.851	2.888	0.574	1.865	0.765	2.141
	Yes		0.867	0.650	2.237	0.830	2.858	0.495	1.572	0.535	1.422
8	No	RF	0.803	0.373	2.234	0.652	3.053	0.290	1.456	0.286	2.696
		XGBoost	0.842	0.479	2.431	0.697	3.201	0.369	1.683	0.425	2.373
	Yes		0.871	0.499	2.133	0.751	3.212	0.361	1.114	0.385	1.411
9	No	RF	0.810	0.341	2.177	0.522	3.263	0.281	1.241	0.284	2.768
		XGBoost	0.860	0.444	2.305	0.647	3.412	0.357	1.348	0.586	2.034
	Yes		0.877	0.427	2.148	0.641	3.075	0.328	1.115	0.326	3.517
10	No	RF	0.704	1.477	5.943	1.500	6.482	1.401	4.970	1.758	6.065
		XGBoost	0.726	1.371	5.510	1.530	6.356	1.168	4.184	1.108	3.622
	Yes		0.729	1.409	5.709	1.573	6.766	1.216	3.640	0.887	6.972
11	No	RF	0.629	1.602	7.290	1.602	7.791	1.536	5.708	2.768	13.966
		XGBoost	0.661	1.418	6.344	1.487	7.461	1.332	4.537	1.645	7.694
	Yes		0.786	1.498	4.749	1.600	5.155	1.360	4.023	1.735	5.062
12	No	RF	0.662	1.685	6.768	1.875	7.395	1.496	6.059	1.919	4.669
		XGBoost	0.679	1.589	6.421	1.825	7.329	1.341	5.358	1.270	3.824
	Yes		0.766	1.450	5.849	1.551	6.024	1.345	5.721	1.038	2.596
13	No	RF	0.618	1.450	8.662	1.939	9.005	1.393	8.268	2.696	8.597
		XGBoost	0.656	1.499	7.795	1.776	8.657	1.246	7.040	1.281	6.323
	Yes		0.758	1.611	6.370	1.874	7.900	1.431	4.602	1.061	2.131
14	No	RF	0.604	1.802	11.684	1.907	13.187	1.722	10.680	2.261	9.304
		XGBoost	0.613	1.673	11.410	1.975	13.151	1.579	10.820	1.653	5.915
	Yes		0.808	1.424	4.603	1.652	5.377	1.177	4.128	0.546	0.537

### Development of myopia and high myopia onset prediction models

Given the promising performance of the XGBoost algorithm in predicting SE, this method was applied to patients with NSE to develop classification models for the onset of myopia and high myopia. In Study 2, baseline characteristics included a mean age of 5.99 ± 2.45 years and SE of 1.72 ± 1.77 D. Similarly, in Study 3, the corresponding values were 9. 24 ± 2.80 years and − 2.12 ± 1.24 D. The likelihood of developing myopia (Study 2) and high myopia (Study 3) during follow-up is 52.71 and 27.10%, respectively. We utilized the same hyperparameter tuning technique used in the original training datasets (Studies 2 and 3) to select the optimal values for the maximum depth, learning rate, and n_estimators, which were determined to be 4, 0.05, and 50, respectively. However, the included data for predicting the onset of myopia showed a moderately imbalanced distribution (1–20%) from the minority class to the majority class in the first and second years (Study 2, Additional file 2: Table S1). Similarly, research on high myopia prediction revealed a moderately imbalanced distribution in the 1–8 years (Study 3, Additional file 2: Table S2). Moreover, to predict myopia onset in the first two years and high myopia onset in 1–8 years, we selected the optimal resampling rates from the lists (0.3, 0.4, 0.5) and (0.7, 0.6, 0.5) for oversampling and undersampling, respectively.

### Myopia onset prediction model performance

**Fig. 4:**
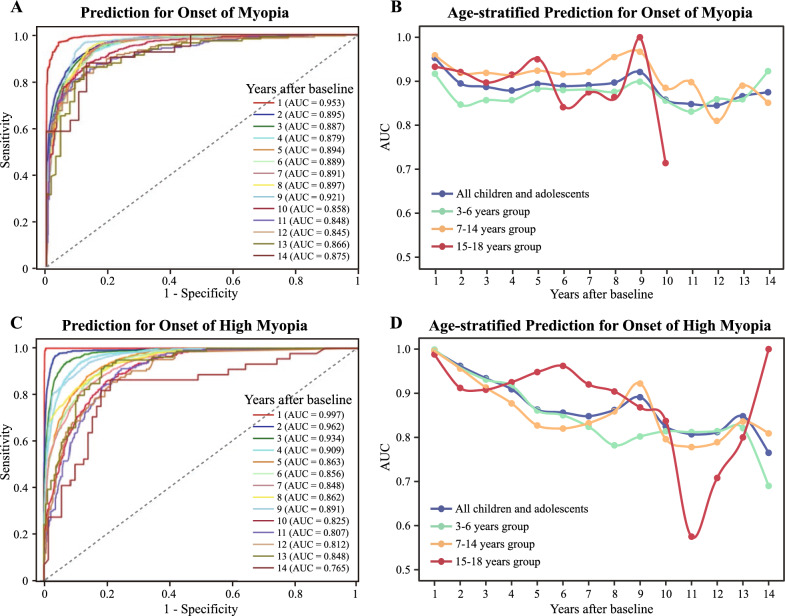
Performance of myopia and high myopia prediction models in testing datasets. ROC curves of the predictive performance for myopia (**A**) and high myopia (**C**) onset from the first to the fourteenth year after baseline. AUC values of the myopia (**B**) and high myopia (**D**) prediction algorithm performance in different time points across different age groups, respectively

Throughout all the prediction years, the performance metrics consistently presented favorable results. For the 15 years of prediction, the AUC, accuracy, precision, sensitivity, specificity, and brier scores were 0.845–0.953, 0.854–0.971, 0.745–0.925, 0.852–0.967, 0.530–0.986, and 0.065–0.181, respectively (Fig. 4A and Additional file 2: Table S1). However, further validation of this model using more extensive data is required to determine the generalizability of this finding. The AUC value was 0.833–0.923, 0.810–1.0, and 0.714–1.0 for the 3–6-, 7–14-, and 15–18-year age cohorts, respectively (Fig. 4B and Additional file 1: Fig. S4A). In the analysis of the 15–18-year group, inspection records were unavailable for the 11–14-year period, hence the AUC was computed for only 10 years. Notably, the predictive performance of myopia tended to be more consistent across different follow-up years in the 3–6- and 7–14-year groups. Conversely, the predictive performance of myopia onset tended to exhibit more fluctuations across different years for 15–18-year group. The detailed predictive performance of the myopia onset model is presented in Additional file 2: Table S1. Additional file 1: Fig. S5 presented calibration curves for predicting myopia onset during the prediction years, revealing consistently excellent classification performance. However, a decline in accuracy is observed in the 10–14 years.

### High myopia onset prediction model performance

The detailed predictive performance of the high myopia onset model is presented in Additional file 2: Table S2. In the testing group, the predictive ability of high myopia onset was comparable to that of the myopia onset prediction algorithm in most prediction years (Fig. 4C). In the first 13 years, sensitivity was consistently higher than precision and F1_scores, with values exceeding 0.8. In particular, in the first four years after baseline, the prediction model for high myopia onset showed high sensitivity, with values exceeding 0.9. Model performance varied significantly within the 3–6-year group, with the AUC ranging from 0.782 to 0.824 at 7–13 years after the initial measurement (Fig. 4D and Additional file 1: Fig. S4B). However, before the sixth year, the AUC consistently exceeded 0.850. In the 7–14-year group, model performance notably dropped in the 10–13-year period, with an AUC of less than 0.8. Nonetheless, in earlier years, the AUC ranged from 0.820 to 0.996. Regarding the 15–18-year group, the AUC values had a limited reference value in the last five years, due to the small amount of available data (n < 20). However, before the ninth year, the AUC remained consistently above 0.868.

### Feature importance in the prediction models

**Fig. 5:**
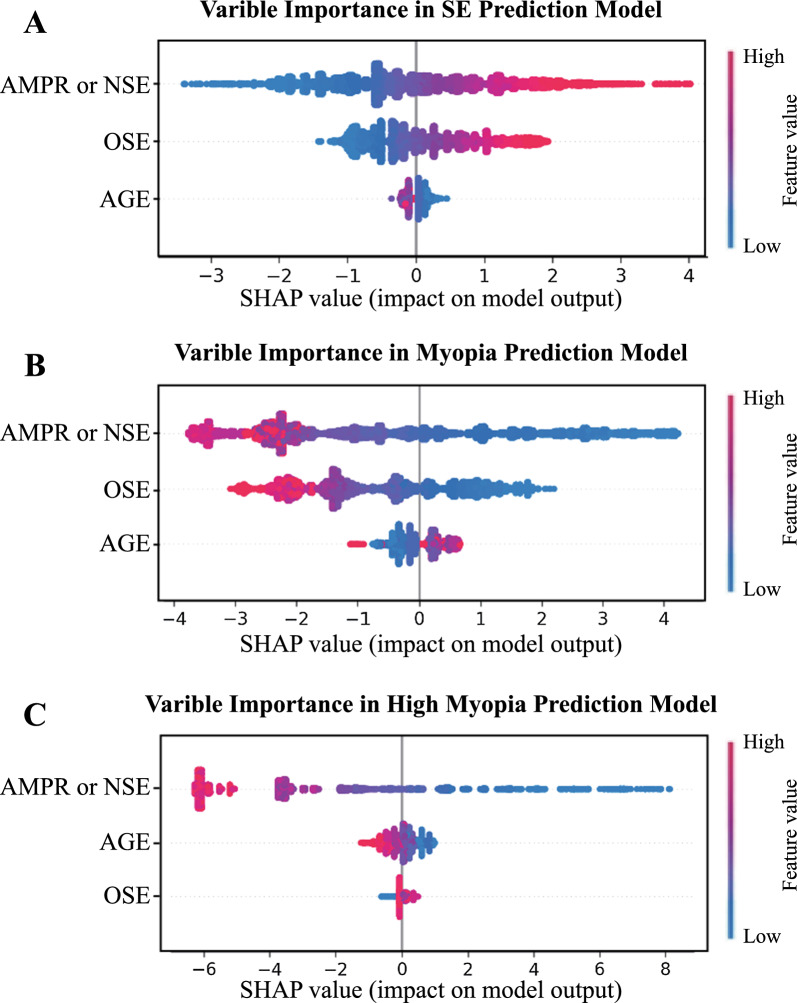
Variable importance analysis across three models. **A**–**C** The scatter plots depict the variable importance for the prediction of SE and onset of myopia and high myopia, respectively. The significance of the feature values varies depending on the variables being analyzed. In the “AMPR” variable, a high value indicates rapid progression of myopia between the first follow-up and baseline, and it is equivalent to NSE in the models. In “OSE” and “NSE”, a high value represents a low diopter, emmetropia, or even hyperopia, while a low value indicates severe myopia. For the “AGE” variable, high and low values correspond to older and younger ages at baseline, respectively

To enhance the interpretability of our predictive models and offer valuable insights for clinical decision-making, we employed the SHAP algorithm to assess the importance of each feature, namely, AGE, OSE, NSE, and AMPR. The results depicted in Fig. 5A, B indicate that the most influential features for the SE and myopia onset prediction models were NSE, AMPR, and OSE. This implies that the initial SE measurement and regular yearly follow-up visits are crucial for accurately predicting the developmental trend of myopia. However, for predicting the onset of high myopia, NSE, AMPR, and AGE were deemed more important than OSE (Fig. 5C). This inspection highlighted the clinical significance of initiating regular visits from an early age, with less emphasis on the initial refractive state, compared to the other two models.

## Discussion

This study investigated the capacity of AI-assisted assessments to advance our understanding of predicting myopia progression, including the onset of myopia and high myopia. We leveraged a dataset comprising numerous participants and a substantial repository of refractory records, which were extracted from electronic medical records and conducted a data- and algorithm-driven investigation. The outcomes facilitated the development of a machine learning model capable of predicting myopia status in Chinese children and adolescents. Our findings provide compelling evidence supporting the adoption of age-specific myopia control strategies in the Chinese population, thereby offering a crucial tool to guide clinical decisions.

In recent years, multiple myopia control treatments with favorable effects have been introduced into clinical practice, such as specially designed spectacle lenses [24], multifocal soft contact lenses [25], orthokeratology lenses [26, 27], and low-dose atropine eye drops [28, 29]. Despite these interventions, not all children effectively manage myopia progression and the onset of high myopia. To enhance clinical outcomes, it is imperative to identify children at the highest myopia risk and administer targeted therapies. Specifically, accurate prediction of onset and progression of myopia based on accessible objective measurements becomes paramount. Our study achieved clinically acceptable predictabilities for SE, myopia onset, and high myopia onset in 3–18 years old children and adolescents, up to 15 years post-initial measurements. Notably, our models demonstrated accurate predictabilities for 7–14-year-old children, a pivotal stage in ocular development and the most susceptible to myopia [30]. Moreover, our study prioritized clinical interpretability by utilizing SHAP algorithm to transparently evaluate feature importance. Specifically, the research underscores the significance of consecutive refractive development records for effective future control measures. Meanwhile, instituting routine early-age follow-ups for children with high myopia risk factors, along with timely follow-ups during the initial stages of myopia in other children, enhances the precision of prediction. This approach enables the identification of high-risk individuals and supports proactive measures for early detection and preventive intervention of myopia.

Methodologically, we selected modeling methods and predictor variables that performed better in the SE prediction models for the prediction of myopia and high myopia onset. For SE predictions using RF algorithm, *R*^2^ consistently exceeded 0.86 for the first nine years after baseline but experienced a significant decline from 10 to 15 years, which partially resulted from the increasing nonlinearity in the dataset [31]. Therefore, we attempted to investigate the added value of a more complex model, the inclusion of an additional predictor, NSE, and an alternative method, XGBoost. The updated model exhibited improved performance, particularly in the last five prediction years. XGBoost, a gradient boosting method, is esteemed for refining machine learning predictions by creating weak models predicting residual errors from prior models during training [32]. In contrast, RF method involves a simple combination of weak trees, each of which provides a prediction and a mode, median, or mean predictive output [33]. RF may exhibit inferior performance compared to XGBoost in certain tasks due to its algorithmic limitation in not explicitly considering inter-factor dependencies, potentially leading to a less effective capture of complex relationships within the data [33]. The enhanced predicted value of the XGBoost signified an increasing non-linear trajectory in myopia development, particularly beyond 9 years. This advantage can be particularly pertinent when integrating complex potential predictors into future analyses for myopia prediction.

For the myopia and high myopia onset prediction, our models presented excellent performance and benefited from resampling and the large-scale sample base. For the prediction of onset of myopia, despite the presence of moderately imbalanced datasets in the first two years, the models' performances remained excellent in the first nine years, with an AUC ≥ 0.879. For the high myopia onset risk prediction, the algorithm exhibited a small drop in classification accuracy compared with the myopia prediction model because of the more severe imbalance ratio of the high myopia class to the non-high myopia class in the real-world population. Similarly, AUC values were > 0.9 for the first four years, and the AUC values ranged from 0.848 to 0.891 for the last nine years. However, in the last five years, the small sample size and more complex non-linearity led to a decrease in the algorithm performance (AUC values ranged from 0.845 to 0.875 and 0.765 to 0.848 in the myopia and high myopia onset prediction model, respectively).

This study has notable strengths, including an extensive sample size and long-term retention of cycloplegic refraction records spanning 15 years. By focusing on three key variables (AGE, OSE, and NSE/AMPR), we successfully achieved effective prediction of the degree of SE and onset of myopia and high myopia for a period of up to 15 years. In comparison to the existing myopia prediction models proposed by Lin [15] (prediction of SE and the onset of high myopia at 18 years of age, as early as eight years in advance, with cycloplegic refraction and annual progression rates) and Li [20] (prediction of myopia progression in primary school children using SE, axial length, and other features), our model achieved comparable predictability in children and adolescents using only three indicators. Therefore, by incorporating two consecutive years of cycloplegic refraction and age, our models could effectively predict SE and the onset of myopia and high myopia. In addition, we developed our models using a diverse age range of 3–18 years, to cater to the clinical demands for myopic consultation and management, which strengthens potential applicability of our AI models in diverse clinical scenarios. Furthermore, through stratified analyses, we discovered that the prediction performance was particularly higher in children aged 3–14 years children than that in 15–18-year-old adolescents, which emphasizes the importance of considering the patient's age when applying the models.

Nevertheless, a recent fundus imaging-based prediction algorithm conducted by Foo [14], concentrating on the 5-year risk of high myopia, attained an average accuracy exceeding 0.9 (AUC value), slightly surpassing our model's performance (AUC: 0.863, prediction accuracy in the fifth year). This outcome underscores the valuable role of fundus examination in predicting myopia progression and high myopia. While our model may not exhibit the same predictive power at a single time point, its relative advantage lies in predicting across multiple time points over an extended duration. Beyond cycloplegic refraction data and fundus imaging, several predictors have been identified to be associated with myopia progression, such as axial length, parental myopia, near work time, and lack of outdoor activity [34, 35]. To achieve a more in-depth understanding of myopia prediction, the exploration of an AI-assisted multimodal model incorporating both “intrinsic” and “extrinsic” factors based on continuous follow-up data presents a promising avenue for future research.

Logically, our models provide a cost-effective means of prediction for accurately predicting and managing childhood myopia. In future clinical practice, the AI model from this study can be seamlessly integrated into mobile terminal apps or clinic medical record systems [36, 37]. By utilizing two consecutive years of cycloplegic refractive measurements, we can dynamically compute the progression of myopia in children, along with the yearly likelihood of myopia or high myopia. This information allows for tailored and timely interventions at key time points, ultimately enhancing overall clinical outcomes.

This study had some limitations. First, there was an evident drop in the algorithm performance for the prediction of SE values after 9 years, and we attempted to update the models from the aspects of potential new input and its combination with another boosting algorithm. However, the reconstructed model's performance did not exhibit significant improvement due to the reduced sample size of individuals followed up for more than nine years. Second, the prognostic power of myopia prediction models for adolescents over 15 years of age may be limited because of insufficient refractive data and the stable nature of myopia progression at this age [38]. Third, data-driven methods necessitate external validation in diverse populations to refine and calibrate the algorithm, enhancing generalizability. Finally, despite achieving balanced and robust performance, concerns about the quality of real-world clinical data and the potential for overfitting issues should be approached with caution.

## Conclusions

This study employed a boosting-based approach called XGBoost to predict the status of myopia among Chinese children and adolescents at specific future time points. Moreover, our findings emphasize the importance of early and regular examinations at a young age for predicting myopia, especially high myopia, Overall, the algorithm presents clinically acceptable accuracy and is straightforward to implement. Our study provides a promising strategy for screening and monitoring myopia status in children and adolescents, and providing myopia interventions in a precise manner.

### Supplementary Information

**Additional file 1: Fig S1.** Spherical equivalent distribution and myopia prevalence for different periods. (A) Spherical equivalent distribution for children and adolescents aged 3 to over 18 years. (B-C) Myopia and high myopia prevalence for individuals included in the study aged 3 to over 18 years. **Fig S2.** Comparative regressive performance of the random forest and XGBoost algorithms for SE prediction. (A) The predictive performance of three models in the whole data set is measured by the goodness of fit (*R*^2^), MAE, and MSE. (B-D) The predictive performance of three models in different age groups measured by MAE and MSE. **Fig S3.** XGBoost-based model performance in predicting SE. Histogram of prediction error (Predicted-Actual) for the first (A) and second (C) prediction year. Scatter plot of predicted and actual values for the first (B) and second (D) prediction year. The black diagonal indicates perfect prediction. **Fig S4.** ROC curves of the performance of the myopia and high myopia prediction algorithms in different age groups. ROC curves of the age-stratified predictive performance for myopia (A) and high myopia (B) onset from the first to the fourteenth year after baseline. Fig. S5 Calibration curves of the performance of the myopia onset prediction algorithms. Calibration curves of the predictive performance for the myopia onset in the training (A) and testing (B) sets from the first to the fourteenth year after baseline.

**Additional file 2: Table S1.** Detailed predictive performance of the myopia prediction model in the testing set. **Table S2.** Detailed predictive performance of the high myopia prediction model in the testing set.

## Data Availability

Data and materials utilized in this study, as well as the associated code, are currently not accessible to the public. However, interested parties may request access from the corresponding author, Prof. Xingtao Zhou and Chencui Huang, through reasonable inquiries.

## Abbreviations

AGE: Age at baseline
AMPR: Annual myopia progression rate
AUC: Area under the curve
AI: Artificial intelligence
*R*^2^: Coefficients of determination
D: Diopters
XGBoost: Gradient boosted tree
IQR: Interquartile range
MAE: Mean absolute error
MSE: Mean squared error
n_estimators: Number of weak learners
OSE: Original SE
RF: Random forest
NSE: SE in the year after the first examination
SHAP: Shapley-Additive-exPlanations
SE: Spherical equivalent
SD: Standard deviation
SMOTE: Synthetic Minority Over-sampling Technique
