# Supplementary material for: Development and validation of predictive models for myopia onset and progression using extensive 15-year refractive data in children and adolescents
Source: J Transl Med. 2024 Mar 17;22:289. doi: 10.1186/s12967-024-05075-0 (PMC10946190; doi:10.1186/s12967-024-05075-0)
Supplement: Supplementary file 1 — Additional file 1: Fig S1. Spherical equivalent distribution and myopia prevalence for different periods. (A) Spherical equivalent distribution for children and adolescents aged 3 to over 18 years. (B-C) Myopia and high myopia prevalence for individuals included in the study aged 3 to over 18 years. Fig S2. Comparative regressive performance of the random forest and XGBoost algorithms for SE prediction. (A) The predictive performance of three models in the whole data set is measured by the goodness of fit (R2), MAE, and MSE. (B-D) The predictive performance of three models in different age groups measured by MAE and MSE. Fig S3. XGBoost-based model performance in predicting SE. Histogram of prediction error (Predicted-Actual) for the first (A) and second (C) prediction year. Scatter plot of predicted and actual values for the first (B) and second (D) prediction year. The black diagonal indicates perfect prediction. Fig S4. ROC curves of the performance of the myopia and high myopia prediction algorithms in different age groups. ROC curves of the age-stratified predictive performance for myopia (A) and high myopia (B) onset from the first to the fourteenth year after baseline. Fig. S5 Calibration curves of the performance of the myopia onset prediction algorithms. Calibration curves of the predictive performance for the myopia onset in the training (A) and testing (B) sets from the first to the fourteenth year after baseline. [file 12967_2024_5075_MOESM1_ESM.docx]

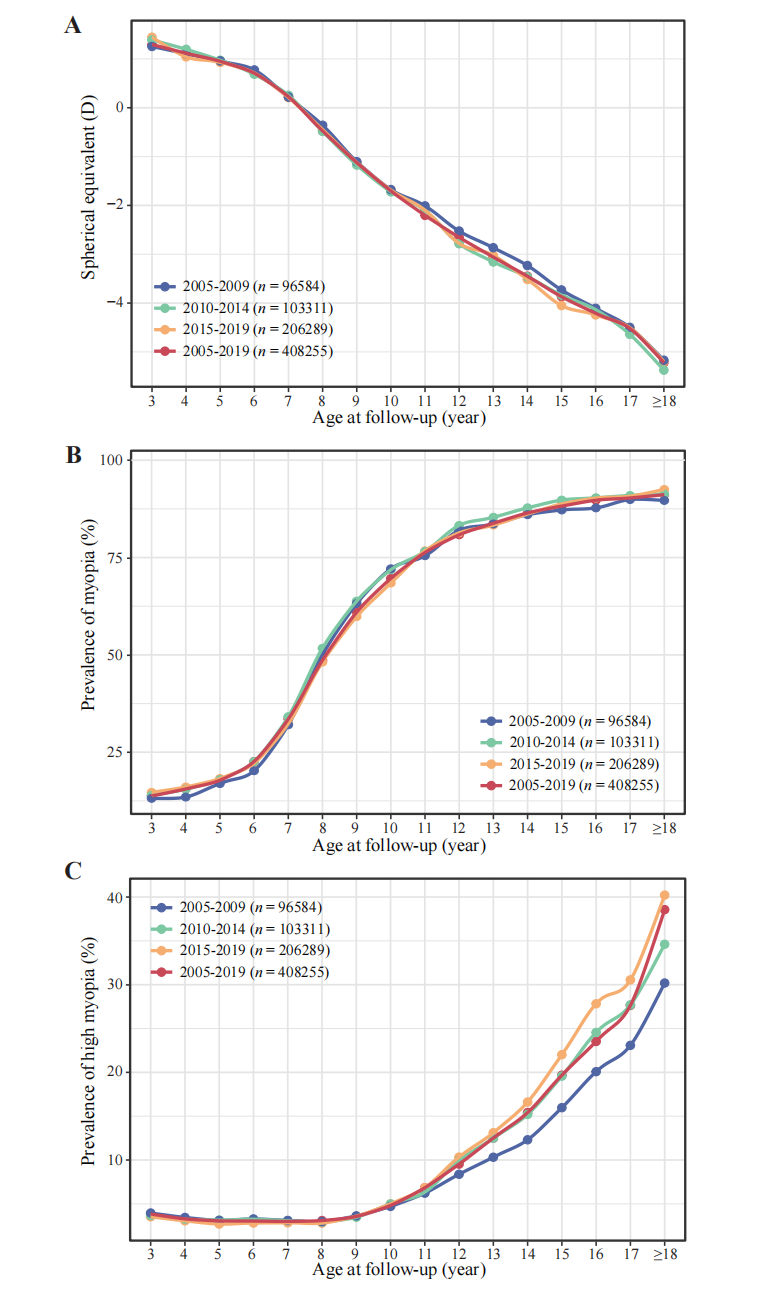


**Supplementary Fig. S1 Spherical equivalent distribution and myopia prevalence for different periods.** (A) Spherical equivalent distribution for children and adolescents aged 3 to over 18 years. (B-C) Myopia and high myopia prevalence for individuals included in the study aged 3 to over 18 years.


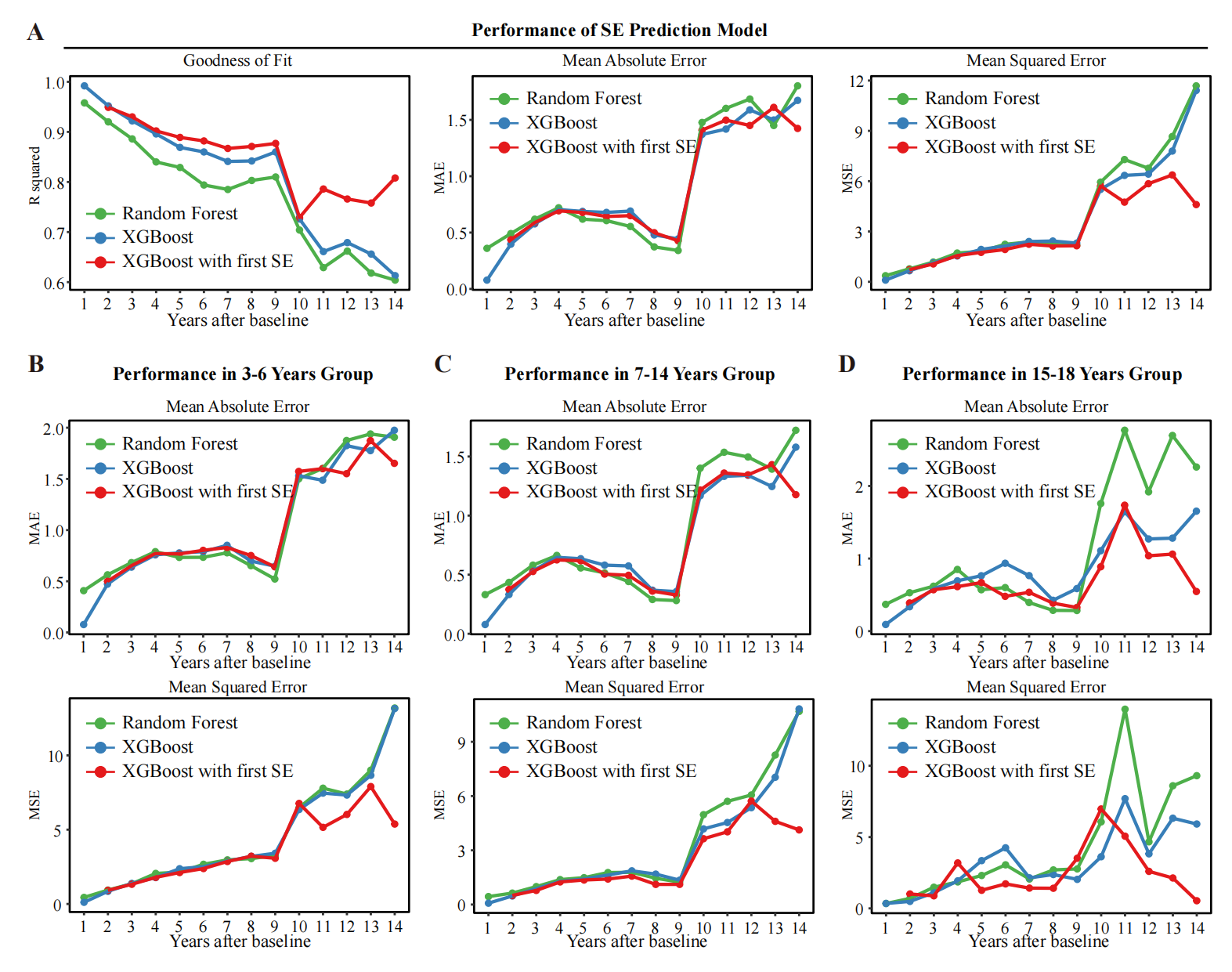


**Supplementary Fig. S2 Comparative regressive performance of the random forest and XGBoost algorithms for SE prediction.** (A) The predictive performance of three models in the whole data set is measured by the goodness of fit (R^2^), MAE, and MSE. (B-D) The predictive performance of three models in different age groups measured by MAE and MSE.


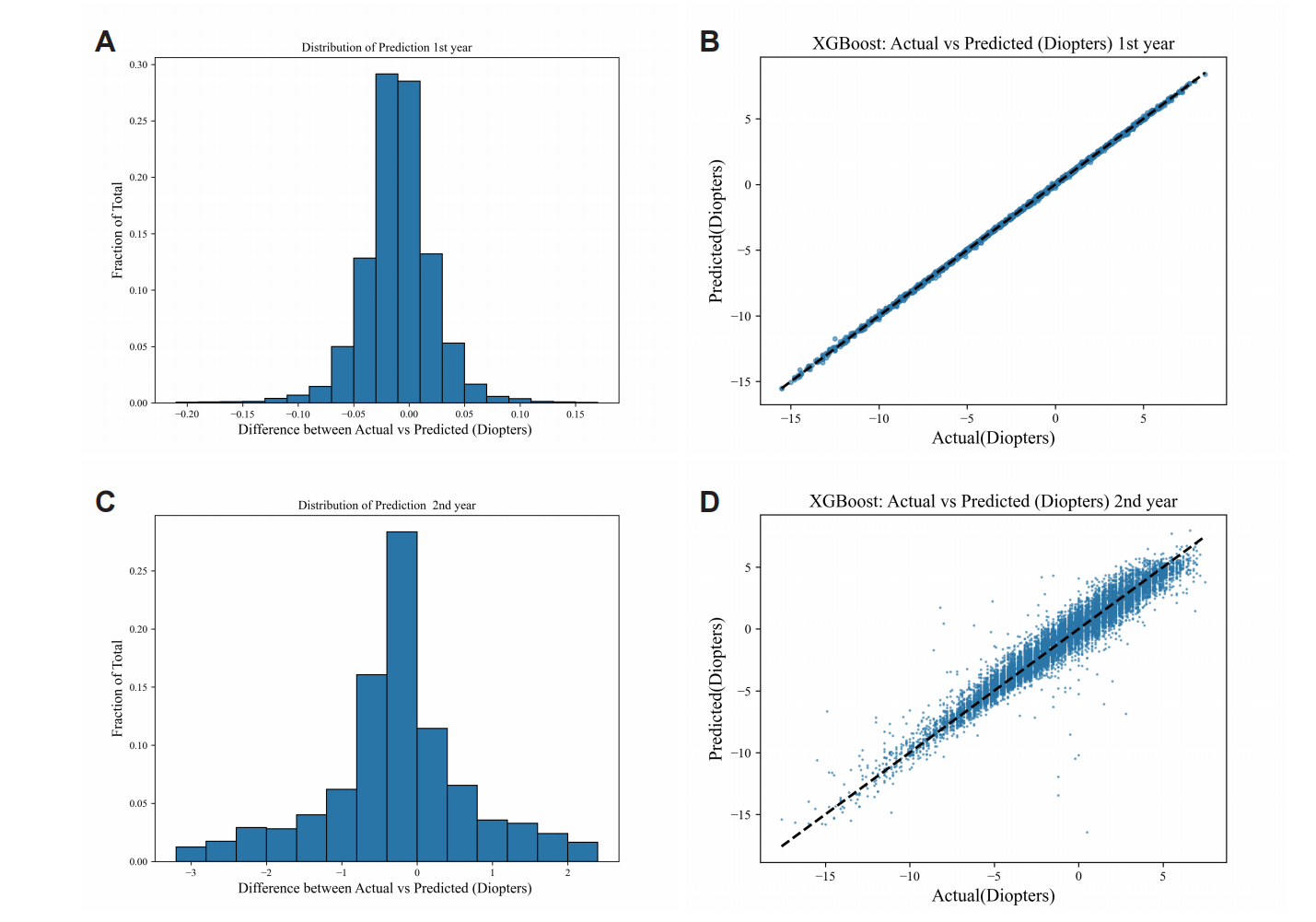


**Supplementary Fig. S3 XGBoost-based model performance in predicting SE.** Histogram of prediction error (Predicted-Actual) for the first (A) and second (C) prediction year. Scatter plot of predicted and actual values for the first (B) and second (D) prediction year. The black diagonal indicates perfect prediction.


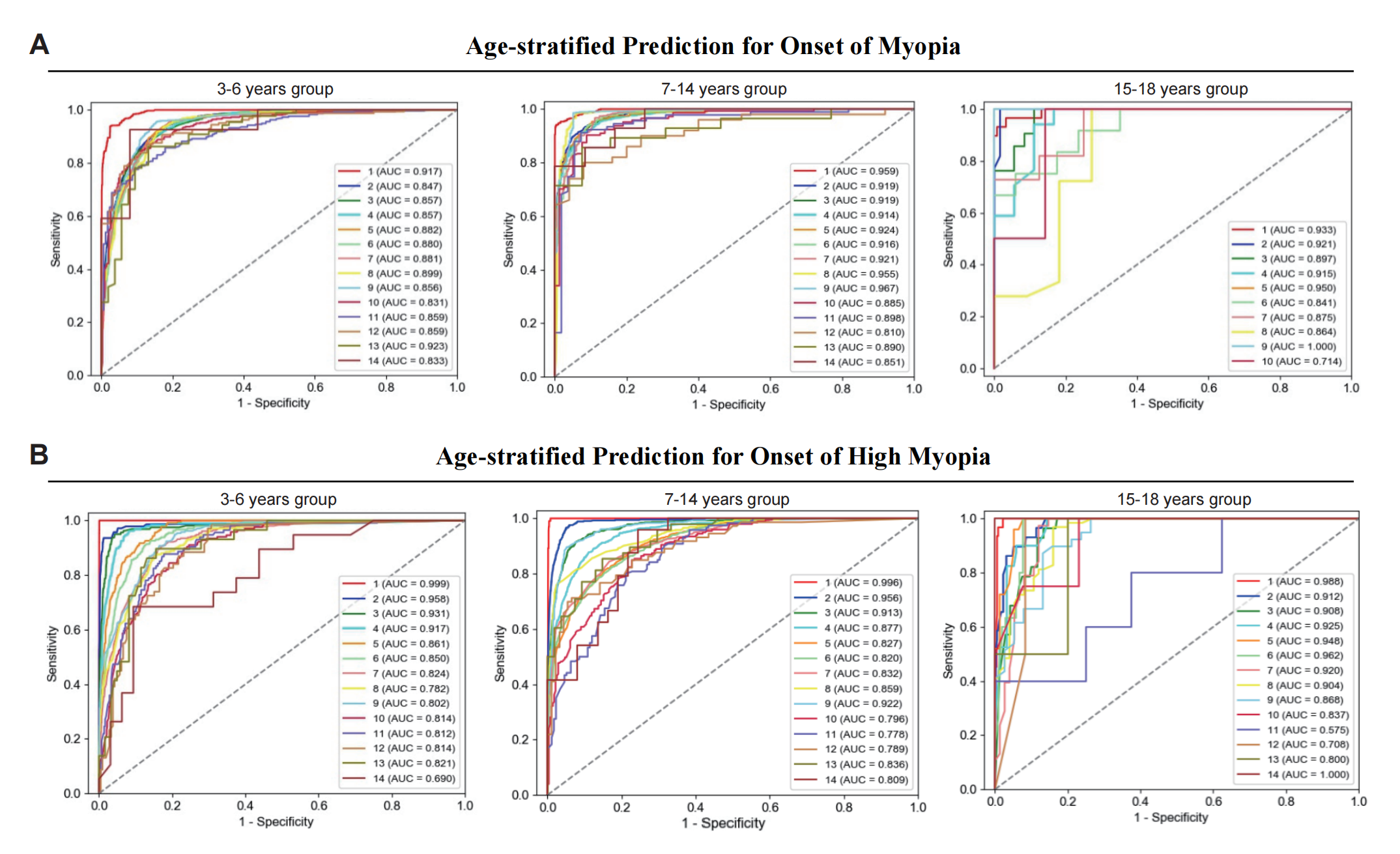


**Supplementary Fig. S4 ROC curves of the performance of the myopia and high myopia prediction algorithms in different age groups.** ROC curves of the age-stratified predictive performance for myopia (A) and high myopia (B) onset from the first to the fourteenth year after baseline.


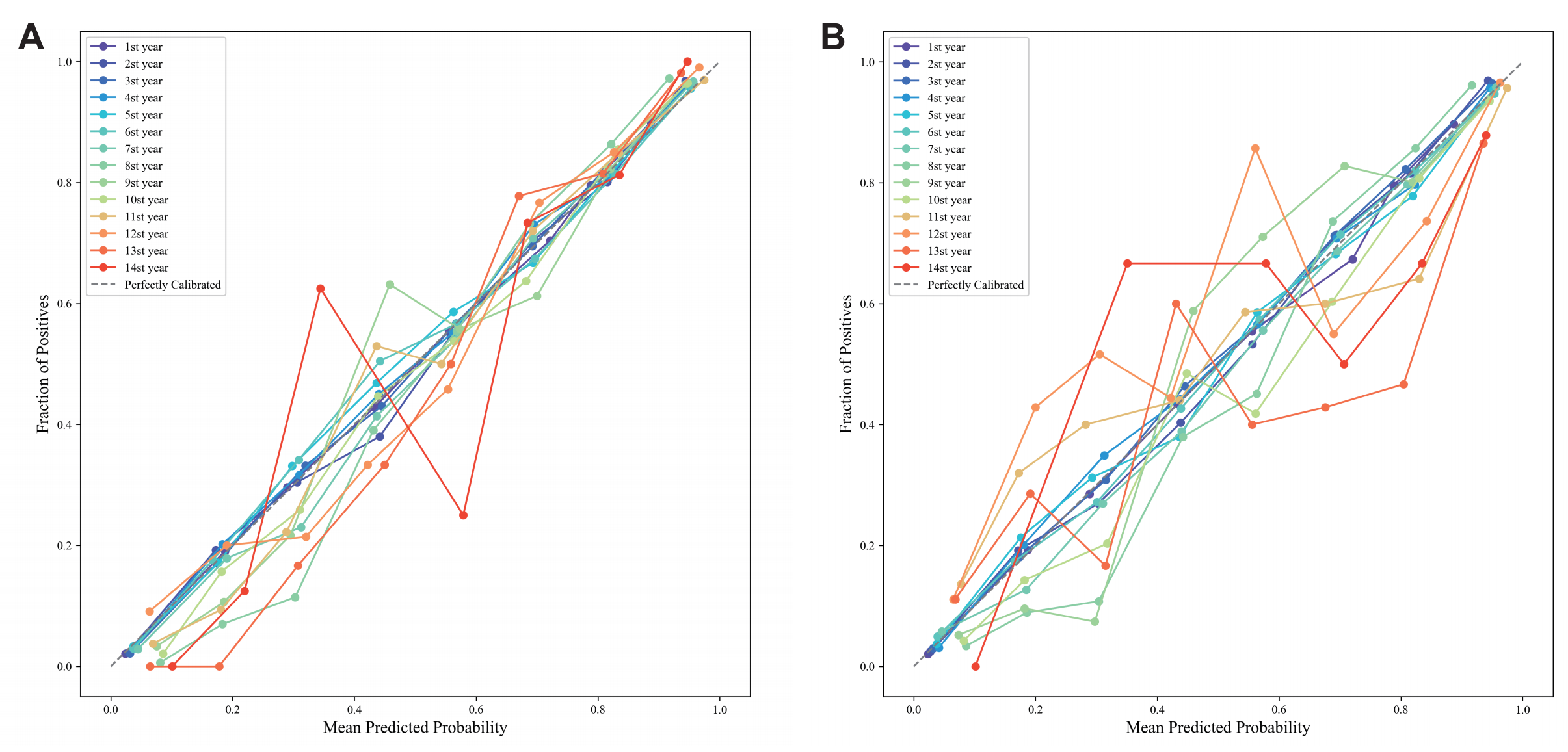


**Supplementary Fig. S5 Calibration curves of the performance of the myopia onset prediction algorithms.** Calibration curves of the predictive performance for the myopia onset in the training (A) and testing (B) sets from the first to the fourteenth year after baseline.
